# Hogging the Air: CAFO Emissions Reach into Schools

**Published:** 2006-04

**Authors:** Julia R. Barrett

Confined animal feeding operations (CAFOs) can pollute the surrounding air with malodorous compounds, bacteria, fungi, and endotoxin. CAFO-related health impacts have been investigated primarily in adults, but children may be at greater risk because of their size and developmental stage. Since children spend considerable time at school, researchers at the University of North Carolina at Chapel Hill conducted an investigation of schools’ proximity to swine CAFOs to determine the extent to which students may be exposed to airborne CAFO emissions **[*EHP* 114:591–596; Mirabelli et al.]**. They determined that some students may encounter CAFO-associated exposures at school and also found that students of color and of low socioeconomic status were the most likely to be affected.

Compared to people living farther away, residents living near CAFOs report a higher prevalence of headaches and respiratory symptoms such as coughing. One study found that CAFO neighbors experience lower secretion and concentration of an immune system protein during odor episodes; another reported livestock odor as having a negative impact on adult levels of tension, depression, and anger. For children, the closer they live to a CAFO, the greater their risk of asthma symptoms.

CAFOs are disproportionately sited in communities of color and areas of poverty. These populations may be more susceptible to the ill effects of airborne exposures owing to existing health challenges such as higher-than-average disease rates and inadequate health care access.

The study findings are based in part on the geographic locations of swine CAFOs and 339 public schools in North Carolina, a state with significant hog, cattle, and poultry industries. Additionally, personnel from 267 schools completed a 21-item survey that included questions about the frequency and intensity of livestock odors in the schools’ indoor and outdoor environments. Publicly available records detailed schools’ racial and ethnic composition and the proportion of students participating in the National School Lunch Program, which provides free or reduced-price meals to students from low-income families. Participation in the program served as an indicator of students’ socioeconomic status.

Of the 226 schools included in the final analysis, distances between a school and the closest swine CAFO ranged from 0.2 to 42.0 miles. Of these, 29% were within 3 miles of one or more swine CAFOs, 21% reported livestock odors outdoors, and 8% reported noticeable livestock odors indoors. The overall average rating of odor intensity was 2.2 on a scale of 1 to 5; the average rating inside buildings was 2.8. Schools with noticeable odors were more likely attended by students of lower socioeconomic status, regardless of race. Schools with more white students or students of higher socioeconomic status tended to be farther from a swine CAFO.

Although the researchers did not characterize the composition of swine CAFO–associated air pollution or identify specific health-related effects, they conclude that livestock-related odors in and around schools may indicate the presence of hazardous airborne contaminants from nearby CAFOs. Their results confirm and expand previous research describing racial and economic disparities in exposure to CAFO emissions.

## Figures and Tables

**Figure:**
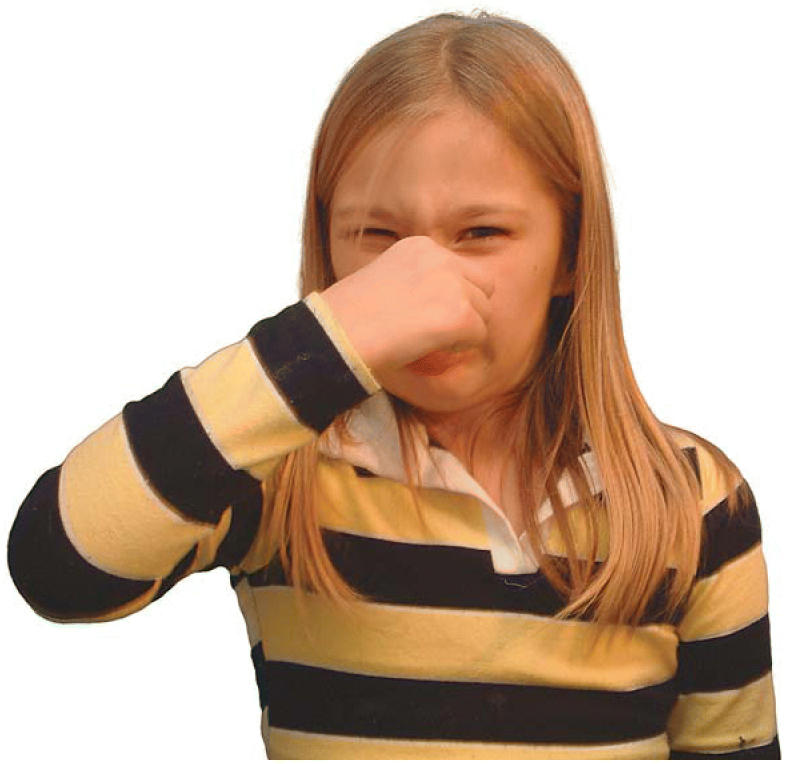
Sniffing out inequalities. New data show that minority and lower-income students are most likely to encounter odors from swine feedlots near schools.

